# The impact of influenza infection on young children, their family and the health care system

**DOI:** 10.1111/irv.12604

**Published:** 2018-09-11

**Authors:** Gabriela A. Willis, David B. Preen, Peter C. Richmond, Peter Jacoby, Paul V. Effler, David W. Smith, Christine Robins, Meredith L. Borland, Avram Levy, Anthony D. Keil, Christopher C. Blyth, the WAIVE Study Team

**Affiliations:** ^1^ Wesfarmers Centre for Vaccines and Infectious Diseases Telethon Kids Institute West Perth Western Australia Australia; ^2^ Department of Health Population Health Services Hobart Tasmania Australia; ^3^ School of Population Health University of Western Australia Crawley Western Australia Australia; ^4^ School of Pediatrics and Child Health University of Western Australia Perth Western Australia Australia; ^5^ Department of General Pediatrics Perth Children’s Hospital Perth Western Australia Australia; ^6^ Department of Health Communicable Disease Control Directorate Shenton Park Western Australia Australia; ^7^ School of Pathology and Laboratory Medicine University of Western Australia Perth Western Australia Australia; ^8^ Department of Microbiology QEII Medical Centre PathWest Laboratory Medicine Nedlands Western Australia Australia; ^9^ Emergency Department Perth Children’s Hospital Perth Western Australia Australia; ^10^ School of Primary Aboriginal and Rural Healthcare University of Western Australia Perth Western Australia Australia; ^11^ Department of Microbiology PathWest Laboratory Medicine Princess Margaret Hospital Nedlands Western Australia Australia; ^12^ Department of Infectious Diseases Perth Children’s Hospital Perth Western Australia Australia

**Keywords:** children, impact, influenza, influenza‐like illness

## Abstract

**Background:**

Influenza is a major cause of respiratory illness in young children. Assessing the impact of infection on children and the community is required to guide immunisation policies.

**Objectives:**

To describe the impact of laboratory‐proven influenza in young children and to compare its impact with that of other respiratory viruses on the child, their family and the health care system.

**Methods:**

Preschool children presenting for care or admission to a tertiary paediatric hospital during the 2008‐2014 influenza seasons were tested for respiratory virus by polymerase chain reaction and culture. Parental surveys were used to determine the impact of infection on illness duration, medication use, absenteeism and health service utilisation. Multivariate regression analyses were used to assess the impact of influenza and to evaluate the association between influenza status and outcomes.

**Results:**

Among 1191 children assessed, 238 had influenza. Among children with influenza, 87.8% were administered antipyretics and 40.9% antibiotics. 28.6% had secondary complications. 65.4% of children missed school/day care, and 53.4% of parents missed work. When influenza and other viruses were compared, significant differences were noted including duration of illness (influenza: 9.54 days, other viruses: 8.50 days; *P* = 0.005) and duration of absenteeism for both the child (23.1 vs 17.3 hours; *P* = 0.015) and their parents (28.5 vs 22.7 hours; *P* = 0.012).

**Conclusions:**

Influenza infection in young children has a significant impact on medication use, absenteeism and the use of health care service. Significant differences are identified when compared with other ILI. These data demonstrate that influenza prevention strategies including immunisation are likely to have wide and significant impacts.

## 1 INTRODUCTION

Influenza is a major cause of respiratory illness in young children,^1^ with those <5 years at greatest risk of disease. The rate of influenza‐associated hospitalisations in this age group exceeds that seen in the elderly.^2^ Assessing the impact of influenza infection in young children is challenging as it co‐circulates with other respiratory viruses, from which it is clinically indistinguishable without laboratory testing.^3^, ^4^ It is well recognised that estimates of influenza morbidity and mortality are likely to be underestimated.^5^

The majority of influenza‐related disease in children is seen in outpatient settings.^1^, ^6^ Despite lower severity, it is speculated that non‐hospitalised influenza represents a greater burden overall.^6^, ^7^ Influenza in children is associated with the frequent use of antipyretics, antibiotics,^6^, ^8^, ^9^ and parental and child absenteeism.^6^, ^10^, ^11^, ^12^, ^13^, ^14^ Health care visits due to influenza illness have been previously estimated to cost the Australian health care system AU$115 million annually (2008).^15^ Indirect costs from parental absenteeism are thought to be the largest contributor to the economic impact of influenza in young children.^10^, ^14^, ^16^

The impact of influenza in children has been well described in the Northern Hemisphere,^6^, ^10^, ^14^, ^17^ yet impact in Australia remains unknown. To date, data on the impact of influenza have been limited to hospitalised children,^18^, ^19^ single influenza seasons^16^, ^18^, ^19^, ^20^ or studies with small numbers of influenza‐positive children.^16^, ^20^

In Australia, influenza vaccine is not included on the National Immunisation Program (NIP), except for Aboriginal and Torres Strait Islander children for whom it was funded in 2015.^21^ Western Australia (WA) introduced a free influenza programme for children aged 6‐59 months in 2008, although uptake rates have been low since safety concerns were identified with one brand of vaccine in 2010.^22^, ^23^ In 2018, following the largest influenza season since 2009, several other Australian jurisdictions introduced influenza vaccine programmes for young children. Prior to inclusion on the NIP, cost‐effectiveness of preschool influenza vaccination needs to be demonstrated. Understanding the impact of paediatric influenza on families and the community is essential to inform such analyses.

Commencing in 2008, the WA Influenza Vaccine Effectiveness (WAIVE) study was established to assess vaccine effectiveness in young children^22^, ^24^, ^25^, ^26^ and to assess the impact of influenza on young children and their families. We describe the impact of laboratory‐proven influenza on the child, their family and health care services, and compare the impact of influenza with other respiratory viruses presenting as influenza‐like illness (ILI).

## 2 METHODS

### 2.1 Study population

As previously described,^24^, ^25^ young children eligible for vaccination (aged 6‐59 months at the time of vaccination) presenting to the emergency department (ED) or admitted with ILI to Princess Margaret Hospital, were eligible for recruitment. Princess Margaret Hospital was the only tertiary paediatric hospital in Perth WA (population 2.5 million people 27), since replaced by Perth Children’s Hospital. ILI was defined as a history of fever ≥37.5°C (by parental report or documented on presentation), plus at least one acute respiratory symptom or sign in the previous 96 hours. Enrolment occurred over seven influenza seasons (2008‐2014), with the season being defined by an increase in statewide influenza detections above baseline. Due to the cohort being part of the wider influenza vaccine effectiveness study, children with a contraindication to influenza vaccination, history of immunosuppression or recent receipt of an immunomodulatory medication were ineligible.

### 2.2 Study procedures

Following written consent, bilateral flocked nasopharyngeal swabs were obtained (Copan Diagnostics Inc. Murrieta, CA). Children from whom a nasopharyngeal aspirate (NPA) was already taken at presentation did not require additional testing. Using previously published methods, samples were tested for respiratory viruses including influenza, respiratory syncytial virus (RSV), human metapneumoviruses, parainfluenza types 1‐4, picornaviruses (including rhinovirus A‐C), human adenoviruses, coronaviruses HKU1/OC43/229E/NL63 and bocaviruses using a polymerase chain reaction (PCR) assay and viral culture.^28^, ^29^, ^30^

Two parent‐completed questionnaires were collected: one at enrolment collecting demographics, comorbidities, school/day care attendance, household composition, passive smoke exposure and influenza vaccination status, and a second questionnaire administered 7‐10 days after enrolment collecting information on the impact of the illness.

### 2.3 Exclusions

Enrolled children for whom a respiratory sample was not successfully collected (n = 16), a second questionnaire was not completed (n = 1946) or a second questionnaire was completed either too early or too late to accurately capture impact data (<6 days or >40 days after enrolment; n = 360) were excluded from the analysis. In addition, to minimise parents reporting the impact from consecutive and/or concurrent viruses not detected by the respiratory sample, children who reportedly had a continuous fever commencing >7 days prior to enrolment were also excluded (n = 102).

### 2.4 Exposure ascertainment and outcomes

Primary exposure was defined as laboratory‐proven influenza A or B by either PCR or culture. Two comparison groups were used: (a) influenza‐negative children and (b) influenza‐negative children testing positive for other respiratory viruses. The second comparison group was used to reduce the probability of including false‐negative influenza infection due to inadequate specimen collection, storage or transport and to exclude children whose symptoms were due to non‐infectious causes.^24^

The outcomes examined were parental reported duration of symptoms (resolution being defined as the majority of symptoms resolving and the child being back to usual activities); prevalence and duration of medication use (over the counter [OTC] and prescription); diagnosis of secondary infections or complications, child absenteeism from playgroup, day care or school; parental absenteeism from work; and health care service use including hospitalisation, visits to general practitioners (GP) or other health care professionals and the performance of diagnostic tests or procedures.

### 2.5 Statistical analysis

Statistical analysis was performed using SPSS^®^ (IBM Corp., Version 22, Armonk, NY) with a level of significance set at *P* < 0.05. Descriptive statistics were performed for baseline characteristics and for all study outcomes. Crude comparisons between children with influenza and other respiratory virus were initially performed using chi‐squared test for categorical variables and independent *t* tests for continuous variables. Separate multivariate regression models were used to evaluate the association between influenza status and study outcomes. Logistic regression was used for binary outcomes and linear regression for continuous variables. All models were controlled for a range of covariates including age, sex, race, comorbidities, prematurity, day care or school attendance >4 hours per week and influenza vaccination status.

The Princess Margaret Hospital for Children Ethics Committee (1673/EP), the South Metropolitan Area Health Service, the Western Australian Aboriginal Health Information and Ethics Committee and the University of Western Australia Ethics Committee (RA/4/1/6456) provided approval for the study.

## 3 RESULTS

**Figure 1:**
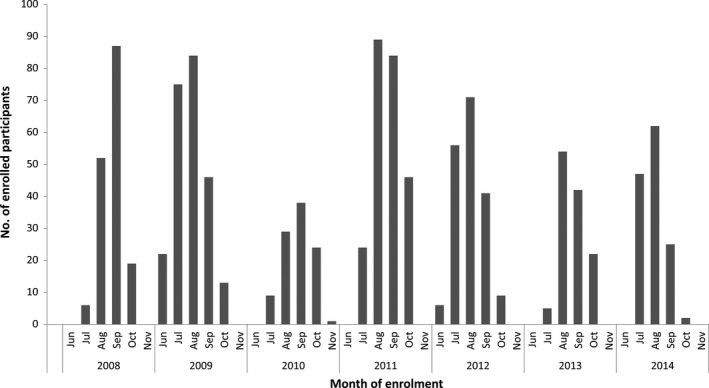
Participant enrolments by month and year, 2008‐14

A total of 1191 children were included in the analysis: 938 ED presentations and 233 hospitalisations. The highest enrolment occurred in 2011 (n = 243) and the lowest in 2010 (n = 101), peaking in August to September each year (Figure 1). Baseline sociodemographic and clinical characteristics of the study sample are shown in Table 1.

**Table 1:** Comparison of demographic and baseline characteristics between influenza‐positive, influenza‐negative and other respiratory virus‐positive children

	All participants (n = 1191)	Influenza‐positive (n = 238)	Influenza‐negative (n = 953)	Other respiratory virus‐positive (n = 670)	Comparison of influenza‐positive and other virus‐positive (*P* value)
Age (y)^a^	2.22 ± 1.27	2.85 ± 1.35	2.06 ± 1.20	1.99 ± 1.15	<0.001
Male gender	690 (57.9)	126 (52.9)	564 (59.2)	397 (59.3)	0.091
Aboriginal/Torres Strait Islander	40 (3.4)	11 (4.6)	29 (3.1)	17 (2.6)	0.114
Preterm (<37 wk)	172 (14.5)	29 (12.4)	143 (15.1)	98 (14.7)	0.380
Any comorbidity^b^	174 (15.0)	31 (13.7)	143 (15.4)	31 (13.7)	0.929
≥4 h childcare/school/wk	741 (62.6)	169 (72.2)	572 (60.3)	402 (60.3)	0.001
No. of adults in household^a^	2.09 ± 0.63	2.12 ± 0.68	2.08 ± 0.62	2.05 ± 0.62	0.253
No. of children in household^a^	1.93 ± 0.95	1.96 ± 0.96	1.92 ± 0.94	1.91 ± 0.93	0.515
Smoker in household	205 (17.5)	38 (16.5)	167 (17.7)	125 (18.8)	0.431
Influenza vaccination status					
Unvaccinated	853 (71.7)	204 (86.1)	649 (68.1)	453 (67.6)	<0.001
Partially vaccinated^c^	100 (8.4)	10 (4.2)	90 (9.4)	68 (10.1)	
Fully vaccinated^d^	237 (19.9)	23 (9.7)	214 (22.5)	149 (22.2)	

a Continuous variables expressed as mean ± SD.

b Including asthma, congenital heart disease, chronic neurological conditions, chronic renal disease, chronic liver disease and inborn errors of metabolism.

c Receipt of current years TIV and at least 2 doses in the current year or in preceding years.

d Receipt of only one dose of TIV in the current year without meeting the definition of fully vaccinated.

**Figure 2:**
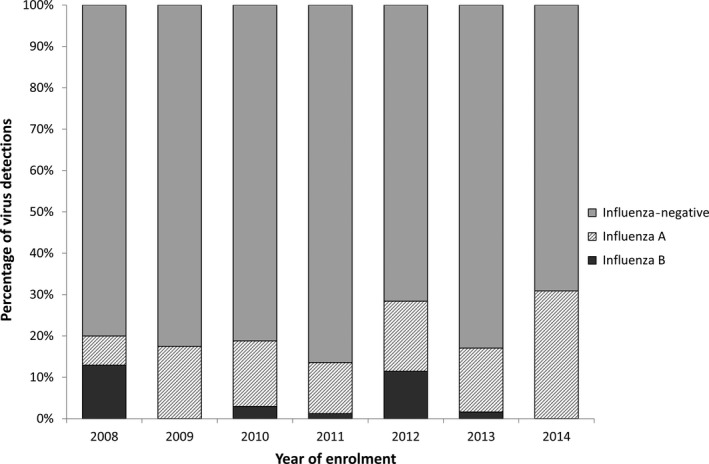
Influenza detections by year (proportion of respiratory samples), 2008‐14

Influenza was detected via PCR in 238 (20.0%), while 953 (80.0%) tested negative for influenza, of which 670 (70.3%) tested positive for another respiratory virus. Influenza detection varied significantly between years (*P* < 0.001). The lowest proportion of influenza positivity was seen in 2011 (13.2%) and highest in 2014 (30.9%; Figure 2).

**Figure 3:**
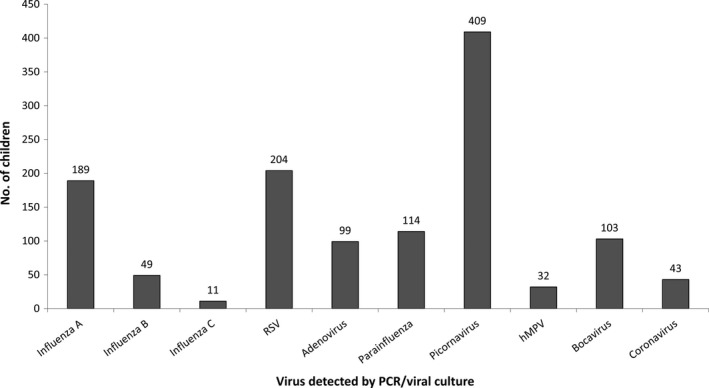
Influenza and other virus detections, 2008‐14

Influenza A/H1, of which the majority was pandemic influenza A/H1N1‐09, was the most commonly detected (n = 117; 49.2% of influenza‐positive samples), followed by A/H3 (n = 72; 30.3%) and influenza B (n = 49 children; 20.6%, Figure 2). Only one child had more than one subtype of influenza isolated from the same sample (A/H1N1‐ 09 and A/H3). The most commonly detected other respiratory viruses were picornaviruses (inclusive of both human rhinoviruses and enteroviruses; n = 409, 34.3%) followed by RSV (n = 204, 17.1%; Figure 3). Co‐infection was common, with 240 children (20.2%) having two viruses detected and 54 (4.6%) having three or more viruses detected. Of children with influenza, co‐infection was observed in 79/238 (33.2%).

Influenza‐infected children were older (mean age 2.85 vs 1.99 years, *P* < 0.001), more likely to be unvaccinated against influenza (86.1% vs 67.6%, *P* < 0.001) and more likely to attend day care or school ≥4 hours a week (72.2% vs 60.3%, *P* = 0.001) compared with children with other respiratory viruses.

### 3.1 Impact of influenza and other respiratory viruses

Impact study outcomes are shown in Tables 2 and 3. As similar results were found, only the comparison between the influenza‐positive and other respiratory virus‐positive groups is shown. The mean duration of influenza illness was 9.54 days, compared to 8.50 days in other respiratory viruses (*P* = 0.005 [Table 3]).

**Table 2:** Categorical variables: number and percentage of children in influenza‐positive, influenza‐negative and other respiratory virus‐positive groups and odds ratio comparing influenza‐positive and other respiratory virus‐positive groups

	Influenza‐positive (n = 238) n (%)	Influenza‐negative (n = 953) n (%)	Other respiratory virus‐positive (n = 670) n (%)	Unadjusted OR (95% CI) (influenza‐positive vs other virus‐positive)	Adjusted OR^c^ (95% CI) (influenza‐positive vs other virus‐positive)
Medication use					
Antipyretics^a^	209 (87.8)	788 (82.7)	551 (82.2)	1.56 (1.01‐2.41)	2.05 (1.24‐3.38)
Decongestant/cough medicine	55 (22.3)	117 (12.4)	80 (12.1)	2.21 (1.51‐3.23)	1.82 (1.19‐2.80)
Antibiotics	97 (40.9)	394 (41.6)	270 (40.6)	1.01 (0.75‐1.37)	0.94 (0.67‐1.31)
Secondary infections					
Pneumonia/chest infection	17 (7.1)	108 (11.3)	70 (10.5)	0.66 (0.38‐1.14)	0.59 (0.33‐1.09)
Febrile convulsion	5 (2.2)	16 (1.7)	9 (1.4)	1.58 (0.52‐4.76)	2.22 (0.61‐8.07)
Ear infection/otitis media	25 (10.8)	94 (10.1)	75 (11.5)	0.93 (0.58‐1.51)	0.90 (0.53‐1.53)
Croup	12 (5.2)	26 (2.8)	13 (2.0)	2.69 (1.21‐5.98)	3.53 (1.43‐8.70)
Any secondary complication	68 (28.6)	273 (28.6)	191 (28.5)	1.00 (0.72‐1.38)	1.04 (0.72‐1.49)
Absenteeism					
Child (school/day care)	155 (65.4)	573 (60.6)	414 (62.3)	0.98 (0.66‐1.45)	0.87 (0.56‐1.35)
Parent (work)	109 (53.4)	449 (55.2)	329 (57.6)	0.84 (0.61‐1.16)	1.02 (0.70‐1.47)
Health service use					
Hospitalisation	38 (16.0)	247 (26.0)	183 (27.5)	0.50 (0.34‐0.74)	0.61 (0.40‐0.93)
≥2 ED visits	45 (18.9)	177 (18.7)	120 (18.0)	1.07 (0.73‐1.56)	1.24 (0.80‐1.90)
GP visit	155 (65.1)	639 (67.2)	455 (68.0)	0.88 (0.64‐1.20)	0.90 (0.64‐1.28)
≥2 GP visits	79 (33.1)	296 (31.1)	221 (33.0)	1.11 (0.77‐1.60)	1.13 (0.76‐1.70)
Paediatrician/other specialist visit	7 (3.0)	60 (6.3)	41 (6.1)	0.47 (0.21‐1.60)	0.58 (0.24‐1.40)
≥2 paediatrician visits	0 (0.0)	23 (2.4)	14 (2.1)	‐	‐
Medical helpline call^b^	56 (23.5)	184 (19.3)	126 (18.8)	1.33 (0.93‐1.89)	1.46 (0.99‐2.16)
≥2 medical helpline calls	16 (6.7)	47 (4.9)	37 (5.5)	0.96 (0.48‐1.93)	1.05 (0.50‐2.22)
Diagnostic tests/procedures					
Blood test	27 (11.4)	105 (11.1)	70 (10.5)	1.10 (0.69‐1.77)	1.18 (0.69‐2.02)
NPA	19 (8.1)	102 (10.2)	77 (11.5)	0.67 (0.40‐1.14)	0.98 (0.55‐1.75)
Chest X‐ray	24 (10.2)	142 (15.0)	100 (15.0)	0.64 (0.40‐1.03)	1.72 (0.43‐1.20)
Lumbar puncture	2 (0.8)	6 (0.6)	2 (0.3)	2.85 (0.40‐20.3)	1.59 (0.12‐20.99)

a Paracetamol and/or ibuprofen.

b HealthDirect or Swine Flu Hotline (2009).

c Adjusted for age, sex, Aboriginal and/or Torres Strait Islander status, any comorbidity, prematurity, attendance at day care/school >4 h/wk and TIV vaccination status.

**Table 3:** Continuous variables: number and percentage of children in influenza‐positive, influenza‐negative and other respiratory virus‐positive groups and linear regression comparing influenza‐positive and other respiratory virus‐positive groups

	Influenza‐positive (n = 238)	Influenza‐negative (n = 953)	Other respiratory virus‐positive (n = 670)	Unadjusted linear regression (influenza‐positive vs other virus‐positive)		Multivariate linear regression^a^ (influenza‐positive vs other virus‐positive)	
				*B* (95% CI)	*P* value	*B* (95% CI)	*P* value
Duration of ILI (d)	9.54 ± 5.28	8.24 ± 4.79	8.50 ± 4.80	1.05 (0.25‐1.84)	0.01	1.26 (0.37, 2.14)	0.005
Duration of medication use (d)							
Paracetamol	5.25 ± 3.04	4.51 ± 3.65	4.54 ± 3.92	0.72 (0.03‐1.40)	0.042	0.90 (0.12, 1.67)	0.023
Ibuprofen	4.91 ± 3.07	4.27 ± 3.09	4.24 ± 3.05	0.68 (−0.02‐1.38	0.058	0.75 (0.01, 1.49)	0.046
Decongestant/cough medicine	5.32 ± 5.41	3.96 ± 2.49	4.17 ± 2.82	1.15 (−0.43‐2.73)	0.153	0.09 (0.03, 0.14)	0.003
Antibiotics	5.89 ± 3.04	7.22 ± 5.57	7.40 ± 6.39	−1.51 (−2.98 to −0.04)	0.044	‐1.09 (−2.63, 0.45)	0.164
Duration of absenteeism (h)							
Child (school/day care)	23.1 ± 19.8	17.3 ± 18.4	17.3 ± 18.4	5.78 (2.29‐9.26)	0.001	4.79 (0.95, 8.63)	0.015
Parent (work)	28.5 ± 27.8	23.0 ± 23.9	22.7 ± 24.1	5.77 (0.26‐11.29)	0.040	8.24 (1.85, 14.62)	0.012
Duration of hospitalisation (d)	2.28 ± 2.33	2.09 ± 1.82	2.10 ± 1.65	0.18 (−0.45‐0.81)	0.571	0.57 (−0.61, 0.74)	0.85

a Adjusted for age, sex, Aboriginal and/or Torres Strait Islander status, any comorbidity, prematurity, attendance at day care/school >4 h/wk and TIV vaccination status.

The majority of children with influenza were given antipyretics (paracetamol and/or ibuprofen; 87.8%) with an average duration of 5.25 days and 4.91 days, respectively. Antibiotics were administered in 41% and decongestant/cough medicine in 22% (Tables 2, 3). Antipyretic and decongestant/cough medicines were used more frequently in children with influenza than with any other viruses (antipyretics: adjusted OR [aOR]: 2.05, 95% CI: 1.24‐3.38; decongestant aOR: 1.82, 95% CI: 1.19‐2.80 [Table 2]). Duration of antipyretic use was significantly longer in children with influenza, but this difference was <1 day of use (paracetamol *B* = 0.90, 95% CI 0.12‐1.67, *P* = 0.023; ibuprofen *B* = 0.75, 95% CI 0.01‐1.49, *P* = 0.046 [Table 3]). No significant difference was seen in either the odds or duration of antibiotic use. Antivirals (eg oseltamivir phosphate) were reported being used in only nine children: six with influenza and three children without.

Overall, 28.6% of influenza‐positive children were reported by parents to be diagnosed with a secondary infection, with otitis media most commonly reported (10.8%). The only significant difference between influenza and other virus‐positive groups was seen in the diagnosis of croup, being significantly higher in children with influenza (aOR: 3.53, 95% CI: 1.43‐8.70 [Table 2]).

Children with influenza were absent from school/day care in 65.4% of cases (mean 23.1 hours or approximately 2.9 days; Tables 2, 3), and parents of these children took time off work to care for the child in 53.4% of cases (mean 28.5 hours or approximately 3.5 workdays). Although no significant differences in the frequency of absenteeism were observed (Table 2), the duration of absenteeism was 4.79 hours longer in those with influenza (Table 3). Similarly, among parents who reported absenteeism, parents of children with influenza missed 8.24 hours more work (95% CI 1.85‐14.62) than parents of children with another respiratory virus (*P* = 0.012; Table 3).

Children with influenza visited ED on average 1.27 times (range: 1‐4 times, SD: 0.64) with 19% requiring more than one visit (Table 2). Two‐thirds of children with influenza were reported to have visited a GP or out of hours GP (Table 2), with over half (51.3%) of those visiting the GP requiring ≥2 visits. No differences were seen in the number and type of health care visit between influenza and other respiratory viruses. Hospitalisation occurred in 16% of influenza‐positive children with an average of 2.28 days (range: <24 hours to 9 days; SD: 2.33; Tables 2, 3). When all children were considered, children with influenza had lower odds than those with other respiratory viruses of requiring hospital admission (aOR: 0.61, 95% CI: 0.40‐0.93). Among children recruited in ED only, there was no significant difference between the proportion being hospitalised (11.6% of influenza‐positive children and 18.6% of other respiratory virus‐positive children; aOR: 0.71, 95% CI: 0.43‐1.17). There were no ICU admissions for children in either group.

Among children with influenza, 26.3% were reported to have a diagnostic test or procedure, including blood tests (11.4%) and chest X‐ray (10.2%). No significant differences were detected between exposure groups for the use of diagnostic tests or procedures (Table 2).

## 4 DISCUSSION

This is the first Australian study to quantify the impact of laboratory‐confirmed influenza infection in preschool children over several consecutive influenza seasons. Our findings illustrate that influenza causes a substantial impact on young children, their families and medical services. Although minor, differences exist between the impact of influenza and other common respiratory viruses presenting as ILI.

Mean duration of influenza illness was found to be 9.5 days, which was approximately 1 day longer than other respiratory viruses. The increased duration of the use of antipyretics and decongestant/cough medicine, and of child and parental absenteeism observed, is likely to be associated with the longer duration of illness.

The use of OTC medicines such as antipyretics, decongestant and cough medicines was frequently administered to minimise the discomfort of influenza, although they are unlikely to have an impact on the course of the illness.^31^ This study demonstrated that the use of antipyretics was very common, being used in 87.8% of children with influenza, for approximately 5 days. This finding is consistent with other studies, with the use of antipyretics in the existing literature ranging from 76.4%^17^ to 99.3%^10^ in influenza‐positive children, albeit with a shorter duration of use than demonstrated here (3.2‐3.9 days, respectively).^10^, ^17^ A lack of awareness or compliance with guidelines on the use of decongestant/cough medicines in children was noted. Decongestant/cough medicines should not be given to children under 2 years.^32^ Nevertheless, such medicines were given to 9% of children with ILI and 14% of children with influenza under 2 years. Similarly in 2013/14, 6% of children with ILI and 8% of children with influenza were given these medicines despite the contraindication being extended to include children under 6 years.^33^

Among children with influenza, 40.9% were treated with antibiotics despite only 42.3% of those treated having a reported bacterial infection. There are significant implications associated with inappropriate prescription of antibiotics, including a significant cost, unnecessary adverse side effects^34^ and the increasing prevalence of antibiotic resistance.^35^ In contrast, the infrequent use of antivirals highlights that empiric antiviral therapy, as opposed to antibacterial therapy, is uncommon in WA, particularly those seen in the emergency department. Of the nine children prescribed oseltamivir, six were shown to have influenza and three did not. Only 13% of children hospitalised with influenza were treated with antivirals. The use of rapid diagnostic tests, which are infrequently used in Australia, may increase the use of antivirals, decrease antibiotic prescriptions and reduce the influenza burden in those children at risk of severe disease and mortality.^36^, ^37^

A substantial social impact of influenza on the family was demonstrated with 65.4% of children having time off school or day care for an average of 3 days, and 53.4% of parents having time off work to care for the ill child. It was also demonstrated that of the children with influenza who attended school or day care, 77.6% were reported to have at least one absence. As the employment status of parents was unknown, it was not possible to determine the proportion of working parents who were absent, but in families with two working parents, the social impact is likely to be greater than in families with one or no working parents. The duration of absence estimate is somewhat higher than in the existing literature, with estimates by Tsolia et al and Principi et al^14^, ^17^ being 1.3 days and 1.2 days. These differences may be attributable to variations in the working patterns or in the virulence of the influenza strains circulating between the populations studied.

Multiple visits to medical practitioners were required in many cases: around two‐thirds of the children with influenza who presented to ED were taken to the GP for the same illness and a third required ≥2 GP visits. In addition, around one‐fifth required two or more ED visits. Those re‐presenting to ED were significantly younger both among those with influenza and overall (data not shown). This represents a high burden on health services with associated economic costs. The rate of hospitalisation for children with influenza and other viruses must be interpreted with caution, as children presenting to ED and hospitalised were both actively recruited. The higher rate of hospitalisation seen with other respiratory viruses was not significant when only children enrolled in ED prior to hospitalisation were included. Despite the limitations, these data are still useful in comparing the impact between influenza and non‐influenza ILI, and also for ascertaining average duration of hospitalisation among children admitted with influenza, which was found to be 2.23 days.

As the study population was limited to children presenting to ED or hospitalised at a single metropolitan tertiary hospital, there are limitations on the generalisability of the findings. Enrolled children had a higher rate of comorbidities and prematurity than the general population. For example, while only 8.5% of babies born in Australia were preterm in 2012,^38^ 14.5% of our sample were reported as preterm. Also, parents of children who were severely unwell may not have been approached by research assistants or less willing to participate, thereby affecting the overall disease severity of the sample. Other limitations included data collection being via parental report rather than through medical record verification, decreasing accuracy of the reported clinical outcomes. Additionally, due to clinical duty of care, parents were informed whether their child was influenza‐positive or influenza‐negative prior to completion of the second questionnaire, which may have affected the reported outcomes.

These data indicate a high impact of influenza illness in WA children and can be used to re‐assess the current economic impact of influenza in young children in Australia.^15^, ^39^

## 5 CONCLUSION

This study demonstrates a significant impact of influenza in young children in WA, in particular showing the high use of antipyretics, antibiotics, common diagnosis of otitis media and high rates of absenteeism for both child and their parents, and the high use of outpatient health care services. In addition, these data demonstrate that influenza has a greater impact than other respiratory viruses causing ILI in terms of the use of antipyretic and decongestant/cough medicines, the diagnosis of croup, the duration of the illness, the duration of absence from school/day care for the child and the duration of absence from work for the parent. Demonstrated preventative strategies, such as universal influenza immunisation in young children, are therefore likely to have a significant impact and should be examined as a priority for inclusion on the Australian immunisation schedule.

## CONFLICT OF INTEREST

Gabriela Willis, Peter Richmond and Christopher Blyth are members of the Vaccine Trials Group, Wesfarmers Centre of Vaccines and Infectious Diseases, Telethon Kids Institute. The Vaccine Trials Group has received funding from vaccine manufacturers for conducting clinical trials, and for travel support to attend immunisation conferences, although not in relation to this study.

Gabriela Willis has received travel support from Pfizer Australia to present the findings of this research at a scientific meeting.

Peter Richmond has previously served on scientific advisory boards regarding influenza vaccines for CSL Ltd. Sanofi, Janssen and GlaxoSmithKline (though has not received personal honorarium), has received travel support from Baxter and GlaxoSmithKline to present at scientific meetings and received institutional funding for investigator‐led research from GlaxoSmithKline and CSL Ltd.

David Smith is a director and board member for the Asia‐Pacific Alliance for the Control of Influenza. It is a not‐for‐profit organisation controlled by an independent board that receives pharmaceutical company funding. He does not receive any payment, only reimbursement of expenses. Prof Smith was a director and board member of a similar organisation, the Australian Influenza Specialist Group, until 36  months ago and has received reimbursement for expenses for attendance at their Annual Scientific Meeting. The other authors have no conflict of interests relevant to this article to disclose.

## AUTHOR CONTRIBUTIONS

Dr Willis assisted in designing the study, cleaned and analysed the data and wrote the first draft of the manuscript. Dr Blyth supervised the project, analysed the data and assisted in writing the manuscript. Professor Preen supervised data cleaning and analysis, and assisted in writing the manuscript. Mr Jacoby assisted in designing the study, supervised the analysis and assisted with writing the manuscript. Professors Effler, Smith and Richmond designed the study, supervised analysis and assisted in writing the manuscript. Ms Robins enrolled participants, supervised research assistants, collated and cleaned the data and assisted with writing the manuscript. Dr Borland supervised the enrolment of participants and assisted with writing the manuscript. Dr Levy coordinated virologic studies, analysed results, collated and cleaned the data and assisted with writing the manuscript. Dr Keil assisted in designing the study, supervised laboratory processing and assisted with writing the manuscript; and all authors reviewed and approved the final manuscript as submitted. All authors approved the final manuscript as submitted and agreed to be accountable for all aspects of the work.

## CLINICAL TRIAL REGISTRATION

None.
